# A Family History of Lethal Prostate Cancer and Risk of Aggressive Prostate Cancer in Patients Undergoing Radical Prostatectomy

**DOI:** 10.1038/srep10544

**Published:** 2015-06-26

**Authors:** Omer A. Raheem, Seth A. Cohen, J. Kellogg Parsons, Kerrin L. Palazzi, Christopher J. Kane

**Affiliations:** 1 Department of Urology, UC San Diego Health System, San Diego, CA, United States

## Abstract

We investigated whether a family history of lethal prostate cancer (PCa) was associated with high-risk disease or biochemical recurrence in patients undergoing radical prostatectomy. A cohort of radical prostatectomy patients was stratified into men with no family history of PCa (NFH); a first-degree relative with PCa (FH); and those with a first-degree relative who had died of PCa (FHD). Demographic, operative and pathologic outcomes were analyzed. Freedom from biochemical recurrence was examined using Kaplan-Meier log rank. A multivariate Cox logistic regression analysis was also performed. We analyzed 471 men who underwent radical prostatectomy at our institution with known family history. The three groups had: 355 patients (75%) in NFH; 97 patients (21%) in FH; and 19 patients (4%) in FHD. The prevalence of a Gleason score ≥8, higher pathologic T stage, and biochemical recurrence (BCR) rates did not significantly differ between groups. On Kaplan-Meier analysis there were no differences in short-term BCR rates (p = 0.212). In this cohort of patients undergoing radical prostatectomy, those with first-degree relatives who died of PCa did not have an increased likelihood of high-risk or aggressive PCa or shorter-term risk of BCR than those who did not.

Prostate cancer (PCa) risk stratification is critical to help physicians and patients decide whether they require treatment and what treatment might be best. Interestingly, family history of PCa, one of the few known risk factors for the disease, is not associated with worse disease at diagnosis or a worse outcome after treatment^1^,^2^. However, the lethality of a patient’s family history, that is whether their first-degree relatives died of the disease, may influence the assignment of PCa risk and fear of adverse outcomes in both patients and physicians^3^,^4^,^5^.

Approximately 10% to 20% of patients with localized PCa are reported to present with a positive family history of PCa. Although it has been clearly described as a finding more common in younger versus older men, there is still significant controversy about the importance of the presence of a positive family history of PCa with respect to presentation and prognosis.

At the genetic level, the association of family history and PCa has been established by characterization of single-nucleotide polymorphisms (SNP) associated with PCa and the recent discovery of the *HOXB13* G84E variant, a germline mutation, associated with increased risk of hereditary PCa^6^,^7^,^8^,^9^. In addition to understanding the link between prevalence and genetics, it would be informative to understand the impact family history of prostate cancer-specific mortality has on the character of the disease process itself.

We sought to determine if lethality of family history (having a first-degree relative die of PCa) is associated with more aggressive PCa clinically or pathologically.

## Patients and Methods

In this study, in which all experimental protocols were approved by the Institutional Review Board of the University of California, San Diego and carried out in accordance with the approved guidelines, we analyzed prospectively collected data from patients undergoing radical prostatectomy, including open and robotic assisted laparoscopic, performed by different surgeons at UC San Diego Health System. We identified three groups: 1) men with no family history of PCa (NFH); 2) a first-degree relative with PCa who had survived the disease (FH); and 3) those with a first-degree relative who had died of PCa (FHD). Death from PCa in the first-degree relatives was confirmed by analysis of the source electronic health record. Informed consent was obtained from all subjects. In addition, individual phone calls were made to patients confirming when cause of death in a first-degree relative was documented as a result of PCa.

Patient demographics, clinical characteristics and prostate cancer risk categories among the three groups included age, race, and body mass index (BMI), use of 5-α reductase inhibitors, comorbidities (hypertension, hyperlipidemia, coronary artery disease, and diabetes), pre-operative prostate specific antigen (PSA), and D’Amico risk stratification. In addition, neoadjuvant and/or concurrent treatment was compared among the three groups (Table 1). The operative outcomes and post-operative complications among the three groups, including total operative time, blood loss, prostate size, use of lymphadenectomy, use of nerve-sparing technique, rate of blood transfusion, length of hospitalization, and rate of post-operative complications are shown in Table 2. The pathologic findings of PCa specimens included tumor size, lymph node yield, Gleason score, T-stage, margins status, perineural invasion (PNI), extensive prostatic intraepithelial neoplasia (PIN), and lymphovascular invasion (LVI); these were compared among the three cohorts. In addition, post-operative outcomes of the 6-week PSA, use of adjuvant treatment, biochemical recurrence, median time to recurrence and median length of follow-up were compared.

Demographic, clinical, and pathologic outcomes were compared using Chi^2^ test, Fisher’s exact test, ANOVA, independent T test (Bonferroni correction for pair comparisons), Kruskal-Wallis test, and Mann-Whitney U test. Biochemical recurrence outcomes were compared using Kaplan-Meier log rank test. Cox logistic regression models were utilized for multivariate analysis to assess for biochemical recurrences. All statistics were performed using SPSS v17.0 (SPSS, Inc., Chicago IL) using two-tailed α = 0.05 as statistically significant.

## Results

Between 2008 and 2011, a total of 600 men underwent radical prostatectomy for organ confined PCa at the hospitals affiliated with our academic institution. However out of this cohort, a total of 471 men had complete database consents and complete family history information and thus were included in this study. Study group populations were: 355 patients (75%) in the NFH group, 97 patients (21%) in the FH group and 19 patients (4%) in the FHD group.

Men in the FH group were diagnosed slightly younger than men in the NFH and FHD groups (p = 0.008). Additionally, more Caucasian men were found in the FH group (84%), compared with the other groups (p = 0.04). Overall, the three groups were similar in most demographics, comorbidities (BMI, DM, hypertension, hyperlipidemia, coronary artery disease, and 5α-reductase inhibitor use) and clinical D’Amico risk stratification. Univariate analysis is shown in Table 1.

With respect to the operative outcomes and post-operative complications, the three groups were comparable with the exception of a higher prevalence of peri-operative blood transfusions in FHD (11%, p = 0.028) (Table 2). On pathologic analysis, the prevalence of Gleason score ≥8 was similar within each group. Similarly, the pathologic T stage was comparable across the three groups. In the NFH group, however, there was higher prevalence of LVI compared to FH and FHD (11%, p = 0.048).

**Figure 1:**
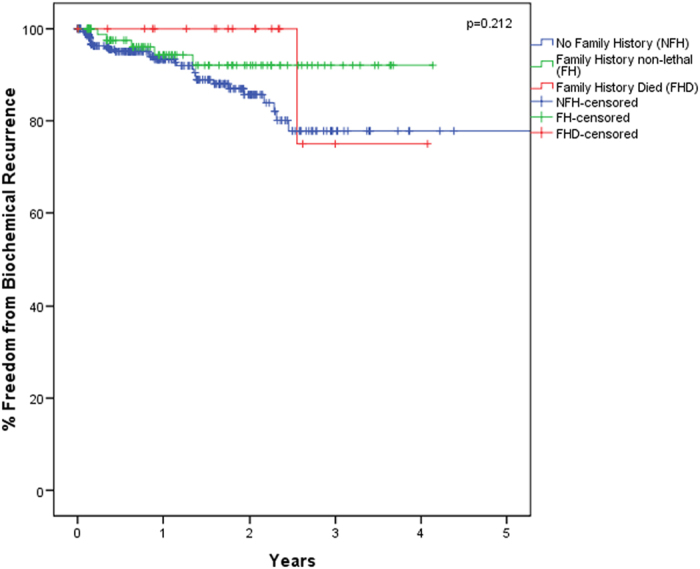
Kaplan-Meier analysis graph for the rate Biochemical Recurrence (BCR) stratified by family history among the no family history (NFH), family history nonlethal (FH) and family history died (FHD) groups.

Biochemical recurrence (BCR) rates were similar for each group: 33/355 patients (9%) in NFH, 5/97 patients (5%) in FH, and 1/19 patients (5%) in FHD (p = 0.376). Median time to BCR and the proportions of adjuvant therapy utilization did not significantly differ between groups. On Kaplan-Meier analysis there were no differences in short-term BCR rates (p = 0.212) (Fig. 1). Overall, the FHD cohort of patients had the longest median follow-up.

Multivariate Cox logistic regression analysis was performed to determine the variables affecting BCR among groups. Table 3 demonstrates that family history of prostate cancer adjusted for age, race and D’Amico risk group did not show significant differences. Furthermore, Table 4 demonstrates that family history of prostate cancer adjusted for age, race, PSA, pathologic Gleason score, pathologic T stage, margin status and LVI did not demonstrate significant differences.

## Discussion

In this prospective study of well-matched radical prostatectomy patients, the NFH, FH and FHD groups had similar demographics, comorbidities and D’Amico Risk stratification, allowing for a meaningful comparison of operative, pathologic, and treatment outcomes. Operative outcomes were similar in almost all respects, including operative time and rate of complication. There was a statistically higher rate of blood transfusions in the FHD cohort; the event rate for blood transfusions was so low in all of the cohorts, however, that this likely has no clinical significance.

To our knowledge, the relationship between lethality of family history of prostate cancer in first-degree relatives and the aggressiveness of the prostate cancer has not been previously investigated in contemporary studies. This cohort demonstrates that there is no association between lethal prostate cancer family history and more aggressive disease of PCa. There was no difference in BCR or adjuvant therapy among the three cohorts. In comparison to the other groups, the NFH group had more LVI (p = 0.048). Furthermore, this finding may suggest that those men with a family history of PCa were potentially more aggressively screened or sought treatment earlier in their disease course. Additionally, this finding appears to lend support to a previous finding by Kupelian *et al.* who, in a systematic analysis of 4,112 patients with stage T1-3 PCa, observed that family history of PCa was not an independent predictor of biochemical relapse^10^. Kupelian’s study has shown that men with a positive family history of PCa presented with more favorable disease and that the overall impact of family history of PCa on prognosis was minimal^10^. Other studies have corroborated the minimal impact family history of PCa has on disease aggressiveness and recurrence^11^,^12^,^13^,^14^.

In this study, we specifically sought to determine if the death of a first-degree relative from PCa, as opposed to just presence of family history, is associated with more aggressive PCa clinically or pathologically. Limitations of this study include a relatively small number of patients with relative short follow-ups for a documented family history of lethal PCa. Although the cohorts are well matched and similar in most of the baseline characteristics, these men were all treated at a tertiary referral center, and there is likely inherited selection bias in this patient population. In addition, although we indicate which patients had primary relatives with PCa specific mortality, we do not know the age at which these family members died – a man who passed away at age 85 from PCa may have had inherently different disease biology from a man who died of PCa in his 60 s. Lastly, although the cohorts within this study are comparable based on baseline demographics and clinical parameters, the results may not be externally applicable in all instances. This population is composed of patients from the West Coast of the United States, particularly Southern California, and may not account for genetic variants abroad.

## Conclusions

In this institutional cohort, patients with a first-degree relative who died of PCa do not appear to have higher-risk, aggressive PCa at diagnosis or a worse outcome after radical prostatectomy, compared to men without a family history or a non-lethal family history of PCa. Future studies with more patients and correlation with specific inherited genetic defects will be critical to fully understand the association of inherited PCa lethality and high-risk, aggressive PCa.

## Additional Information

**How to cite this article**: Raheem, O. A. *et al.* A Family History of Lethal Prostate Cancer and Risk of Aggressive Prostate Cancer in Patients Undergoing Radical Prostatectomy. *Sci. Rep.* **5**, 10544; doi: 10.1038/srep10544 (2015).

## Figures and Tables

**Table 1:** Patients’ demographic, clinical characteristics and prostate cancer risk stratification among the three groups.

Variables	No Family History Prostate Cancer (NFH)	Family History Prostate Cancer Non-Lethal (FH)	Family History Prostate Cancer Died (FHD)	p-value
	n = 355 (75%)	n = 97 (21%)	n = 19 (4%)	
Age ± SD, mean (years)	62 ± 6.7	60 ± 7.4	61 ± 7.6	0.008*
Race				0.041*
Caucasian	252 (71%)	81 (84%)	13 (69%)	
Other	103 (29%)	16 (17%)	6 (32%)	
BMI ± SD, mean (Kg/m2)	27.7 ± 4.2	27.5 ± 3.7	27.2 ± 4.7	0.831
Hypertension	142 (40%)	36 (37%)	5 (26%)	0.454
Hypercholesterolemia	132 (37%)	32 (33%)	3 (16%)	0.140
Diabetes Mellitus (DM)	29 (8%)	6 (6%)	1 (5%)	0.747
Coronary artery disease	19 (5%)	6 (6%)	2 (11%)	0.625
5α-reductase inhibitor (Proscar/Avodart)	24 (7%)	3 (3%)	1 (5%)	0.397
Pre-operative PSA (ng/mL) (IQR), median	5.9 (4.2–8.6)	5.3 (4.1–6.6)	5.7 (3.7–6.6)	0.094
Clinical T Stage				0.759
T1a-c	226 (64%)	67 (70%)	12 (67%)	
T2a-c	120 (34%)	28 (29%)	6 (33%)	
T3a-b	8 (2%)	1 (1%)	0	
Biopsy Gleason Score				0.16
≤ 6	153 (45%)	55 (59%)	9 (50%)	
7	115 (34%)	23 (25%)	7 (39%)	
≥ 8	70 (21%)	15 (16%)	2 (11%)	
D’Amico risk group				0.099
Low risk	143 (40%)	52 (54%)	6 (32%)	
Intermediate risk	133 (38%)	28 (29%)	10 (53%)	
High risk	79 (22%)	17 (18%)	3 (16%)	
Neoadjuvant/Concurrent treatment	21 (6%)	3 (3%)	0	0.314

SD, standard deviation; BMI, body mass index; PSA, prostate specific antigen.

^*^statistically significant (p < 0.05).

**Table 2:** Operative outcomes and post-operative complications among the three groups.

Variables	No Family History Prostate Cancer (NFH)	Family History Prostate Cancer Non-Lethal (FH)	Family History Prostate Cancer Died (FHD)	p-value
	n = 355 (75%)	n = 97 (21%)	n = 19 (4%)	
Median operative time (IQR), (minutes)	188 (160–210)	185 (160–209)	180 (166–207)	0.892
Median EBL (IQR), (mL)	150 (100–200)	150 (100–200)	175 (100–200)	0.591
Median prostate weight (IQR), gm	48 (38–60)	47 (39–48)	42 (38–48)	0.210
Peri-operative blood transfusion (units)	11 (3%)	0	2 (11%)	0.028*
Lymph nodes dissection (node)	190 (54%)	44 (45%)	7 (37%)	0.161
Nerve sparing technique				0.319
None/Partial	68 (20%)	12 (13%)	3 (17%)	
Complete	281 (81%)	82 (87%)	15 (83%)	
Median hospital stay (IQR), (days)	1 (1-1)	1 (1-1)	1 (1-1)	0.471
Post operative complications	85 (24%)	23 (24%)	6 (32%)	0.745
Low grade complications	75 (21%)	20 (21%)	4 (21%)	0.994
High grade complications	23 (7%)	4 (4%)	2 (11%)	0.500

EBL, estimated blood loss.

^*^statistically significant (p < 0.05).

**Table 3:** Multivariate logistic regression for biochemical recurrences adjusting for family history prostate cancer, age, race, and D’Amico risk group.

Covariates	Hazard ration (HR)	95% Confidence Interval (CI)		p-value
		Lower	Upper	
**Family history prostate cancer**				.250
Non-Lethal	.520	.200	1.354	.181
Lethal	.331	.045	2.457	.280
**Age**	.976	.931	1.024	.324
**Race** (non-Caucasian)	.487	.211	1.120	.090
**D’Amico Risk Group**				<0.001*
Intermediate risk	4.135	1.129	15.145	.032*
High risk	19.723	5.837	66.639	<0.001*

^*^statistically significant (p < 0.05).

**Table 4:** Multivariate logistic regression for biochemical recurrences adjusting for family history prostate cancer, age, race, PSA, surgery Gleason score, pathologic T stage, margin status and lymphovascular invasion.

Covariates	Hazard ration (HR)	95% Confidence Interval (CI)		p-value
		Upper	Lower	
**Family history prostate cancer**				.370
Non-lethal	.492	.178	1.358	.171
Lethal	.646	.084	4.986	.676
**Age**	1.009	.962	1.059	.701
**Race** (non-Caucasian)	.350	.135	.908	.031*
**Prostate specific antigen (PSA)**				.008*
PSA 10–19	3.520	1.586	7.813	.002*
PSA 20+	2.109	.676	6.581	.199
**Pathologic Gleason Score**				.017*
Gleason 7	1.582	.333	7.525	.564
Gleason 8–10	5.468	1.113	26.852	.036*
**Pathologic T stage**				.070
pT3	1.605	.638	4.039	.315
pT4	6.839	1.295	36.127	.024*
**Positive margins**	1.892	.874	4.098	.106
**Lymphovascular invasion (LVI)**	2.547	1.072	6.051	.034*

^*^statistically significant (p < 0.05).

## References

[b1] Lin K., Croswell J. M., Koenig H., Lam C. & Maltz A. Prostate-specific antigen-based screening for prostate cancer: an evidence update for the U.S. Preventive Services Task Force. In Evidence Syntheses, No. 90 Report No. 12-05160-EF-1 (Agency for Healthcare Research and Quality (US), 2011 Oct). 22171385

[b2] Slomski A. USPSTF finds little evidence to support advising PSA screening in any man. JAMA 306, 2549–2551 (2011). 22187266 10.1001/jama.2011.1804

[b3] Cerhan J. R. *et al.* Family history and prostate cancer risk in a population-based cohort of Iowa men. Cancer Epidemiol Biomarkers Prev. 8, 53–60 (1999). 9950240

[b4] Johns L. E. & Houlston R. S. A systematic review and meta-analysis of familial prostate cancer risk. BJU Int. 91, 789–794 (2003). 12780833 10.1046/j.1464-410x.2003.04232.x

[b5] Bratt O. *et al.* Effects of prostate-specific antigen testing on familial prostate cancer risk estimates. J Natl Cancer Inst. 102, 1336–1343 (2010). 20724726 10.1093/jnci/djq265

[b6] Zheng S. L. *et al.* Cumulative association of five genetic variants with prostate cancer. N Engl J Med. 358, 910–919 (2008). 18199855 10.1056/NEJMoa075819

[b7] Xu J. *et al.* Inherited genetic variant predisposes to aggressive but not indolent prostate cancer. Proc Natl Acad Sci USA 107, 2136–2140 (2010). 20080650 10.1073/pnas.0914061107 PMC2836698

[b8] Ewing C. M. *et al.* Germline mutations in HOXB13 and prostate-cancer risk. N Engl J Med. 366, 141–149 (2012). 22236224 10.1056/NEJMoa1110000 PMC3779870

[b9] Kupelian P. A., Kupelian V. A., Witte J. S., Macklis R. & Klein E. A. Family history of prostate cancer in patients with localized prostate cancer: anindependent predictor of treatment outcome. J Clin Oncol. 15, 1478–1480 (1997). 9193343 10.1200/JCO.1997.15.4.1478

[b10] Kupelian P. A. *et al.* Aggressiveness of familial prostate cancer. J Clin Oncol. 24, 3445–3450 (2006). 16849760 10.1200/JCO.2006.05.7661

[b11] Azzouzi A. R. *et al.* Familial prostate cancer cases before and after radical prostatectomy do not show any aggressiveness compared with sporadic cases. Urology 61, 1193–1197 (2003). 12809896 10.1016/s0090-4295(03)00033-5

[b12] Roehl K. A., Loeb S., Antenor J. A., Corbin N. & Catalona W. J. Characteristics of patients with familial versus sporadic prostate cancer. J Urol. 176, 2438–2442 (2006). 17085123 10.1016/j.juro.2006.07.159

[b13] Rouprêt M. *et al.* Outcome after radical prostatectomy in young men with or without a family history of prostate cancer. Urology 67, 1028–1032 (2006). 16698363 10.1016/j.urology.2005.11.035

[b14] Siddiqui S. A. *et al.* Impact of familial and hereditary prostate cancer on cancer specific survival after radical retropubic prostatectomy. J Urol. 176, 1118–1121 (2006). 16890705 10.1016/j.juro.2006.04.077

